# Infant with Known Dandy–Walker Malformation and Poor Feeding Found to Have Additional Diagnosis

**DOI:** 10.1055/a-2562-1814

**Published:** 2025-04-08

**Authors:** Jacob Q. Lin, April Cooke, Nick Townley

**Affiliations:** 1 Department of Pediatrics, Creighton University, School of Medicine, Omaha, Nebraska

**Keywords:** Dandy–Walker malformation, Noonan syndrome, prenatal diagnosis

## Abstract

**Background:**

There are few reported cases of Dandy–Walker Malformation associated with Noonan syndrome (NS).

**Case presentation:**

We herein present a case of a late preterm infant with Dandy–Walker malformation (DWM) that underwent a workup for feeding difficulty and was found to have NS. This is one of the few reported cases of DWM with NS having a PTPN11 gene mutation.

**Conclusion:**

Overlapping clinical features may disguise diagnosis in infants with multiple pathologies.

## Background

There have been very few reported cases of Dandy–Walker malformation (DWM) associated with Noonan syndrome (NS). The first case reported in 2022 was that of a newborn with PTPN11 mutations. 1 Another case was that of a child with DWM who was diagnosed with NS at 16 years old due to further investigations for concerns of their short stature and delayed puberty. 2 A recent case study also described a 5-year-old patient who was diagnosed with NS (PTPN11 mutation) with Chiari malformation type I and syringomyelia. 3 In addition, another case report by Weinstock described a patient with NS and a pathogenic RRAS2 variant (p.Q72L), which is a different mutation from the one described herein. The patient described by Weinstock exhibited severe features, including low-set, posteriorly rotated ears, redundant nuchal skin, widely spaced nipples, and cryptorchidism. 4 The present case is one of only a few cases of a patient with DWM and NS. This case is unique as the NS was found incidentally during genetic testing.

NS is a genetic disorder that presents with a wide range of severities and manifestations. Familial cases are consistent with those of autosomal dominant inheritance. However, the majority of cases are inherited via de novo mutations. Owing to the extent of variable expressivity, the incidence of NS is difficult to determine as many individuals with mild manifestations remain undiagnosed. Nevertheless, the disorder is fairly common, with the severe phenotype affecting 1:1,000–1:2,500 live births. 5 NS is often diagnosed early when there is an affected parent; otherwise, the age at diagnosis can have a broad range. Typical facial features include hypertelorism, epicanthic folds, broad forehead, and low-set posteriorly rotated ears. 6 The most common heart defect affecting 50 to 60% of patients is pulmonary valve stenosis. Although the birth weight and length of patients are often normal, 63% of them fail to thrive due to feeding difficulties. 7 NS can be diagnosed based on clinical features alone, though molecular genetic testing will be confirmatory. 5

DWM is a congenital anomaly that mainly affects the prenatal development of the cerebellum and is the most common cause of posterior fossa malformation. 8 This malformation occurs when the fourth ventricle foramina and Blake's pouch (the roof of the rhombencephalon) do not form correctly; a normal formation is completed by 18 weeks of gestation. 8 DWM is often diagnosed during second-trimester ultrasound, which would demonstrate ventriculomegaly and/or enlarged cisterna magna. 8 9 A “classic” triad exists, including complete or partial agenesis of the vermis; enlarged posterior fossa with an upward displacement of the tentorium, transverse sinus, and torcular; and cystic dilation of the fourth ventricle. The most common early manifestation is hydrocephalus, which occurs in approximately 80% of cases. 9

## Case Presentation

The patient is a male neonate with a gestational age of 36 weeks and 2 days. He was born to a 36-year-old multiparous mother. The mother has had three total pregnancies, two full-term and one preterm (referring to the present case). All of the children are alive. The medical history of both parents was noncontributory, and neither was diagnosed with developmental delays.

**Fig. 1:**
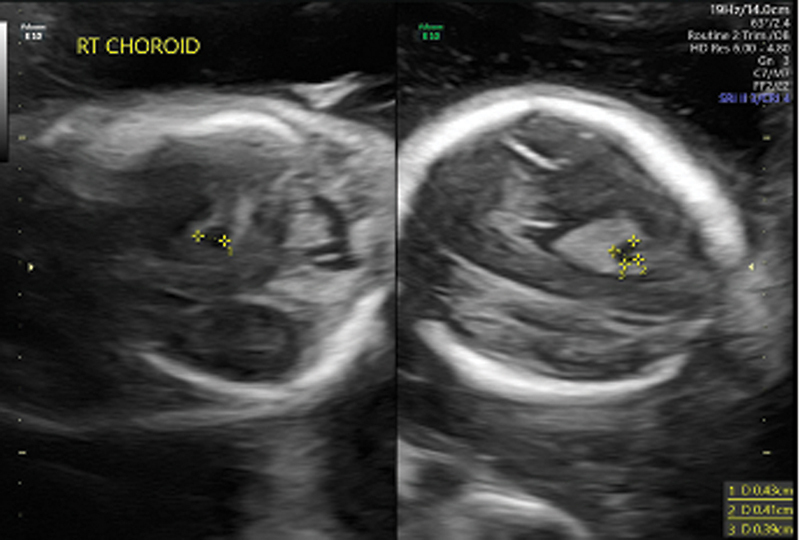
Ultrasound images at gestational week 20 showing right choroid plexus cyst measuring 0.43 × 0.41 × 0.39 cm.

The first-trimester sonographic evaluation at 8 weeks indicated a grossly normal developing singleton intrauterine pregnancy. During a transabdominal ultrasound monitoring of fetal anatomy at 20 weeks, a right choroid plexus cyst was observed, with no other gross anatomic abnormalities Fig. 1A .

**Fig. 2:**
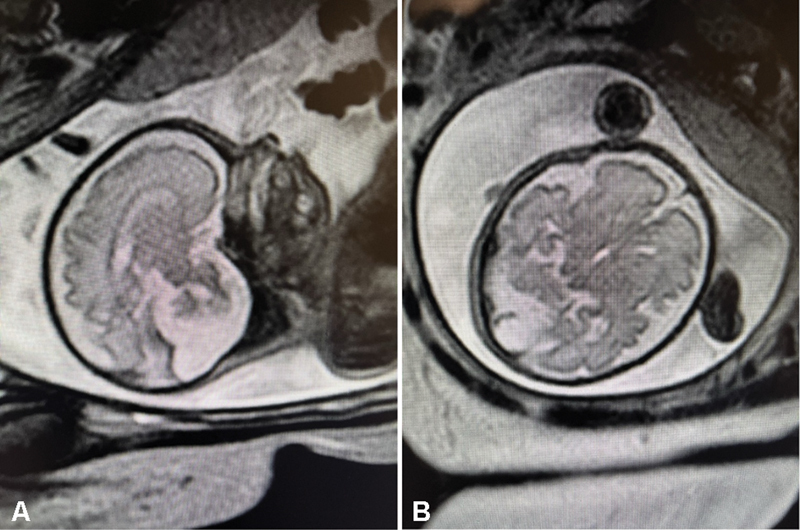
( **A** ) 31-Week sagittal MRI showing cerebellar vermis hypoplasia; the fourth ventricle communicating with an enlarged retrocerebellar cerebrospinal fluid space. ( **B** ) 31-Week MRI, coronal image for mother, and axial cut for fetus.

A subsequent ultrasound performed at 28 weeks revealed a normal choroid plexus cyst; however, an elevated amniotic fluid index value of 37 cm was observed, indicating polyhydramnios. The patient was then closely followed by maternal–fetal medicine (MFM). At 29 weeks, ultrasound-guided amnioreduction was performed. A magnetic resonance imaging (MRI) performed at 31 weeks confirmed the sonographic findings, in addition to cerebellar vermis hypoplasia, communication of the fourth ventricle with an enlarged retrocerebellar cerebrospinal fluid space, and torcular elevation Fig. 2A and B .

MFM performed an amniocentesis and repeated amniodrainage at 32 weeks. At the 32-week visit, an MFM ultrasound revealed an enlarged cisterna magna and dilated fourth ventricle, which suggested DWM. Cell-free DNA testing showed a low risk for trisomies 21, 18, and 13. After the amniocentesis, genetic analysis was conducted via array comparative genomic hybridization, which was negative for genomic imbalances. A total of five amnioreductions were performed throughout the pregnancy for symptomatic polyhydramnios.

**Fig. 3:**
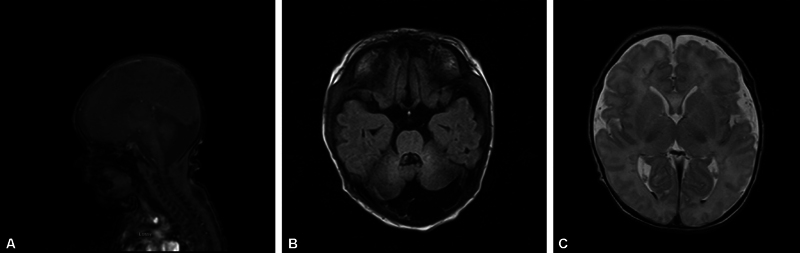
( **A** ) Day of life (DOL) six sagittal MRI, brain with cerebellar/vermian hypoplasia and dilated fourth ventricle. ( **B** ) DOL six axial MRI, cerebellar/vermian hypoplasia compatible with Dandy–Walker malformation spectrum. ( **C** ) Axial MRI showing dark T2 signal blood products in the dependent portions of the bilateral occipital horns.

At 36 weeks and 2 days, the patient's mother presented to the hospital in labor. The mother normally delivered (vaginal) an infant with a birthweight of 2,885 g (58%) and a length of 47 cm. The Apgar scores were 1 and 7 at 1 and 5 minutes, respectively, and 2 minutes of PPV and CPAP were required before weaning off in the neonatal intensive care unit. Genetic consultation was conducted on the day of life (DOL) 2. On DOL 6, the MRI was repeated, which revealed DWM with small areas of infarction in the cerebral white matter and small intraventricular hemorrhage Fig. 3A–C .

Concerns for seizure-like activity prompted an EEG, which revealed no evidence of such activity but showed abnormal spikes in multifocal areas, indicating cortical irritability. The workup included an echocardiogram, sonographic spinal imaging, renal ultrasound, and ophthalmic examination, all of which yielded unremarkable results. At this time, CH was normally progressing as a premature baby, and his clinical status was complicated by oral feeding difficulties. Attempts to ad lib feedings were made, but the patient was not able to maintain adequate oral intake. Therefore, a decision was made to conduct GeneDx genome sequencing.

One month later, the genetic testing showed a positive PTPN 11 gene mutation for NS. The parents were also tested but were negative for the aforementioned mutation, suggesting that the case involved a de novo rather than an inherited mutation. The clinicians did not observe any abnormal features typical of NS.

However, the NS diagnosis shed some light on the patient's oral feeding difficulties. On DOL 35, the decision was made to place a gastrostomy button to ensure proper nutrition and appropriate weight gain.

## Discussion

To our knowledge, this is the second reported case of a neonate diagnosed with DWM and NS with a de novo heterozygous PTPN11 mutation. However, other reports have described cases of NS with the PTPN11 gene variant that are complicated by Chiari malformation type I and/or syringomyelia. 3 For this patient, the specific variant is c.417 G > C p.(E139D), with an autosomal dominant mode of inheritance. DWM is associated with chromosomal abnormalities or syndrome in 33% of cases, cardiovascular conditions in 27%, and eye and ear diseases in 24%. 10 NS is characterized by postnatally reduced growth, facial dysmorphic features (hypertelorism, ptosis, and low-set ears), congenital heart defects, skeletal abnormalities, and webbed neck. 7 Feeding problems occur in 65 to 76% of patients with NS, with approximately 38% of them requiring feeding tubes. 11 12 The present case is unique as the patient was incidentally diagnosed with NS when genetic testing was ordered to investigate potential genetic abnormalities associated with DWM and feeding difficulties. The patient lacks typical dysmorphic facial and anatomic abnormalities, except for a dilated fourth ventricle and cerebellar vermis hypoplasia. This absence is not necessarily atypical as it has been reported that many individuals may remain undiagnosed throughout their lives. 13

At this time, it is difficult to speculate the extent to which this patient will be affected by NS and DWM throughout his life. The feeding difficulties associated with DWM and NS were likely exacerbated by the presence of the other. Although research is limited, a study involving 108 patients with NS reported a significant improvement in feeding difficulties between the ages of 1 and 2, with only a few of them continuing to have feeding difficulties after the age of 2 years. 14 Although knowledge of this diagnosis does not introduce a disease-specific treatment, awareness of the presence of NS allows the medical team and family to understand the etiology of feeding difficulties and shape expectations.

In addition to feeding difficulties, the co-occurrence of NS and DWM can manifest with more severe neurological manifestations. In DWM, the primary neurologic complication is hydrocephalus. In addition, patients with DWM have an increased risk of cognitive impairment and epilepsy compared with health controls. NS also increases the risk of neurological changes, with 94% of patients having visual/ocular problems, 50% having hypotonia, and 13% having recurrent seizures. 15 The patient in the present case was diagnosed with DWM via prenatal ultrasound, which revealed an enlarged cisterna magna and dilated fourth ventricle. Although the patient did not demonstrate significant neurological disabilities, it is difficult to predict what neurologic complications he will face given the combined risk for neurological disabilities as he has two concomitant disorders. Notably, his EEG did not demonstrate seizure-like activity but showed cortical irritability, which could increase his chance of developing seizures in the future. Therefore, clinical monitoring and close follow-up are in place.

## Conclusion

In conclusion, our case shows a possible correlation between DWM and NS. This case emphasizes how overlapping clinical features can complicate diagnosis and management. Overall, demonstrating the importance of early diagnosis encourages adequate interdisciplinary support and monitoring. Future studies and case reports are warranted to better understand the etiology of this association and improve patient outcomes.
